# The Effect of Detectable HIV Viral Load among HIV-Infected Children during Antiretroviral Treatment: A Cross-Sectional Study

**DOI:** 10.3390/children5010006

**Published:** 2018-01-01

**Authors:** Visal Moolasart, Suthat Chottanapund, Jarurnsook Ausavapipit, Sirirat Likanonsakul, Sumonmal Uttayamakul, Don Changsom, Hatairat Lerdsamran, Pilaipan Puthavathana

**Affiliations:** 1 Bamrasnaradura Infectious Diseases Institute, Ministry of Public Health, 11000 Nonthaburi, Thailand; suthat_97@yahoo.com (S.C.); charen977@gmail.com (J.A.); siratlik3@gmail.com (S.L.); katesumonmal@gmail.com (S.U.); 2 Department of Microbiology, Faculty of Medicine Siriraj Hospital, Mahidol University, 10700 Bangkok, Thailand; don_custom@hotmail.com (D.C.); Hatairat.lerd@gmail.com (H.L.); Pilaipan.put@mahidol.ac.th (P.P.)

**Keywords:** detectable viral load, HIV-infected children, ILI, poor adherence, lower nadir CD4

## Abstract

The RNA viral load of human immunodeficiency virus (HIV) is initially used to determine the status of the HIV infection. The goal of therapy following treatment failure is to achieve and maintain virologic suppression. A detectable viral load may relate to the progression of HIV infection. A cross-sectional survey was conducted from January 2013 to December 2014 at the Bamrasnaradura Infectious Diseases Institute, Thailand. The aim was to determine the prevalence of detectable HIV viral load (dVL) and analyze the factors associated with post-dVL conditions that occur independently of a switch to a new antiretroviral agent. The prevalence of dVL was 27% (27 of 101). The mean ages of dVL and non-dVL children were 12.0 and 12.3 years, respectively. Age, sex, body mass index for age *z*-scores, previous tuberculosis disease history and parental tuberculosis history of both groups were not significantly different (*p >* 0.05). The prevalence of poor adherence (<95%), influenza-like illness (ILI) and opportunistic infections were higher in dVL than non-dVL children (*p <* 0.05). The mean nadir CD4 cell count during the study was lower in dVL than non-dVL children (646 compared to 867, respectively; *p* < 0.05). Other factors were not significant (all *p* > 0.05). In multivariable analysis, dVL was significantly associated with ILI (odds ratio (OR) = 9.6, 95% confidence interval (CI) = 1.3–69.4), adherence (OR = 0.195, 95% CI = 0.047–0.811) and nadir CD4 during the study (OR = 1.102, 95% CI = 1.100–1.305). The prevalence of dVL was 27% with this dVL among HIV-infected children found to be associated with ILI, poor adherence and lower nadir CD4 during the study.

## 1. Introduction

Highly active antiretroviral therapy (HAART) reduces mortality and morbidity among human immunodeficiency virus (HIV)-infected children [1,2,3]. Appropriate HAART, immunizations and prophylactic antimicrobials for HIV-infected individuals have been demonstrated to substantially reduce mortality and prevent hospitalizations [4]. Achieving the World Health Organization (WHO) 2020 treatment goals and the goal of stopping the acquired immune deficiency syndrome (AIDS) epidemic as a public health threat by 2030 [5] will depend on the success of current HIV treatment programs. The success will not only depend on access to HIV treatment, but also on good adherence to antiretroviral therapy (ART) and retention in care. This is necessary to achieve viral suppression, prevent viral failure, diminish viral transmission and reduce HIV/AIDS-related deaths [6]. Some children may be taking ART, but these agents are not controlling their HIV. Poorly controlled HIV can be due to many factors, including lack of health care, poor medication adherence, incomplete drug absorption, drug resistance and drug toxicity. Treatment failure can be clinical failure, immunologic failure, virologic failure or any combination of the three. The goal of therapy following treatment failure is to achieve and maintain virologic suppression as measured by a plasma viral load below the limits of detection using the most sensitive assay [7]. Non-detectable viral load or virologic suppression is defined as having a plasma viral load below the lower level of detection using highly sensitive assays with lower limits of quantitation of 20–75 copies/mL [7]. Although HAART is very effective at reducing viral loads to undetectable levels, the immunological function of patients does not fully recover to pre-HIV levels. Therefore, immunocompromised individuals still have a significantly higher likelihood of infection by other pathogenic viruses (e.g., influenza virus) and experience worse symptoms compared with healthy persons [8]. There are several infectious conditions, such as influenza/influenza-like illness (ILI), lower respiratory tract infections (LRTIs), opportunistic infections (OIs) and tuberculosis (TB) in HIV-infected children.

Influenza-like illness and influenza are major public health threats in HIV-infected and non-HIV-infected children. Influenza vaccination is the most appropriate way to prevent ILI and influenza infection in comparison to placebo or no interventions [9]. Lower respiratory tract infections in HIV-infected children are from common childhood respiratory viral, bacteria and opportunistic pathogens, such as *Pneumocystis jirovecii*, cytomegalovirus (CMV) and *Mycobacterium tuberculosis* (MTB) [10,11,12,13,14]. Poor general health, malnutrition, immune depletion, chronic lung disease, increased household exposure and vaccine responsiveness may all contribute to the risk of LRTI. A wide range of pathogens causing LRTIs and pneumonia in children with HIV has been reported [15]. Opportunistic infections are infections that occur more frequently and are more severe in individuals with weakened immune systems, including people with HIV. Opportunistic infections are caused by a variety of viruses, bacteria, fungi and parasites. All of the countries with a TB incidence of more than 500 cases per 100,000 population are in sub-Saharan Africa, although large case numbers have also been found in Asian countries with large populations [16]. A previous study demonstrated that TB is associated with a high mortality rate when it is diagnosed in the context of virologic failure [17]. In addition, it was previously shown that having a father as a main caregiver and lack of access to tap water are risk factors for viral failure [18]. Distance from clinic results in loss to follow up, with good access to care associated with closer distance to clinic, which was defined as living ≤40 km from the clinic site [19].

Human immunodeficiency virus RNA viral load is used initially to determine the status of early HIV infection and to monitor the disease. Detectable viral load (dLV) may relate to the progression of the HIV infection. The aim of the present study is to determine the prevalence of dVL and analyze the factors associated with post-dVL condition, independently of switching to a new antiretroviral agent (ARV).

## 2. Materials and Methods

### 2.1. Study Design

We performed an observational study in a cohort of HIV-infected children. All children were originally infected with HIV through vertical transmission. The study was conducted from January 2013 to December 2014 in HIV-specialized pediatric wards of the Bamrasnaradura Infectious Diseases Institute (BIDI). Data from January to December of 2014 were collected prospectively, while data before 2014 were collected retrospectively.

This study was reviewed and approved by the Ethical Committee for Research in Human Subjects of the Department of Diseases Control, Ministry of Public Health, Nonthaburi, 11000, Thailand and by the institutional review board of the Bamrasnaradura Infectious Diseases Institute, Bamrasnaradura Infectious Diseases Institute, Ministry of Public Health, Nonthaburi, 11000, Thailand. The reference approval letter codes are SO12h/58 and 11/55-562.

The BIDI is a tertiary hospital under the Ministry of Public Health in Nonthaburi, Thailand. Our services include health services, infection control, training and research. In particular, we care for all aspects of HIV disease in HIV-infected children, such as ART, treatment failure, virologic failure, vaccines and other infections. In this study, the annual influenza vaccination for all HIV-infected children was funded by the universal health coverage system of Thailand, with the influenza vaccines given every 12 months.

The primary study objective was to determine the prevalence of dVL among HIV-infected children during ART. The secondary study objective was to analyze the factors associated with post-dVL condition, regardless of switching to a new ARV. The inclusion criteria were as follows: (1) HIV-infected children; (2) age of 18 years or less; (3) receiving ART for at least 2 years; and (4) follow-up at least three times during a period of 12 months. The exclusion criteria were as follows: (1) visiting other hospitals; and (2) loss to follow up. 

We compared basic demographics and characteristics at the first visit as well as the clinical and laboratory factors during this study between dVL and non-dVL children. We collected data at the first viral load result with sex, age and body mass index for age *z*-scores, which included body mass index (BMI) for age *z*-scores (standard deviation scores), previous TB disease history and parental TB history. All children were classified into two groups: (1) detectable viral load was defined as VL ≥ 50 copies/mL; and (2) non-detectable viral load children was VL < 50 copies/mL. We observed each case for one year after the viral load result. Data including the proportion of adherence ≥95%, last WHO stage, HAART (protease inhibitor (PI) or non-PI), nadir CD4 cell count, nadir hematocrit level, peak cholesterol, peak triglyceride, hospitalization history, pneumonia, ILI, OIs, study status, distance of residence, caregiver type and having a father as the main caregiver (yes or no) were collected.

We have an ILI center and illness surveillance was coordinated by self-reporting and telephone call reporting during the follow-up period. Influenza-like illness was defined as a sudden onset of symptoms at least one of four systemic symptoms (fever/feverishness, malaise, headache or myalgia) and at least one of three respiratory symptoms (cough, sore throat or shortness of breath) [20]. Pneumonia was defined clinically as having a cough or tachypnea and dyspnea or chest retractions [21] without the presence of OIs within the lungs. Opportunistic infections was defined as most common opportunistic infections, such as: (1) candidiasis of bronchi, trachea, esophagus or lungs; (2) invasive cervical cancer; (3) coccidioidomycosis; (4) cryptococcosis; (5) chronic intestinal cryptosporidiosis of greater than one month’s duration; (6) cytomegalovirus: CMV (particularly retinitis); (7) HIV-related encephalopathy; (8) herpes simplex virus (chronic ulcer(s) with greater than one month’s duration, bronchitis, pneumonitis or esophagitis); (9) histoplasmosis; (10) chronic intestinal isosporiasis with greater than one month’s duration; (11) Kaposi’s sarcoma; (12) lymphoma (multiple forms); (13) TB; (14) disseminated or extrapulmonary *Mycobacterium avium* complex, *Mycobacterium kansasii* or other Mycobacterium; (15) *Pneumocystis carinii* pneumonia; (16) recurrent pneumonia; (17) progressive multifocal leukoencephalopathy; (18) recurrent Salmonella septicemia; (19) toxoplasmosis of brain; and (20) wasting syndrome due to HIV [22]. Hospitalization was defined as at least one hospitalization to the BIDI during the period of follow-up. Short and long distances of residence were defined as living ≤40 km and >40 km from the study center, respectively [19]. Interpretation of cut-offs for BMI for age *z*-scores (standard deviation scores) were overweight: >+1 standard deviation (SD); obesity: >+2 SD; thinness: <−2 SD; severe thinness: <−3 SD; and recommended: from −2 SD to + 1 SD [23,24,25].

A questionnaire was filled out by the children or parents. Adherence to ART was defined as the regular monthly collection of prescribed ART from the pharmacy of the clinic. Each refill period was identified as the interval between the last pharmacy visit date and the scheduled refill date. Refill adherence was 100% if all pills during the scheduled refill period were collected on time. Refill percent values above 100% for patients who refilled earlier than scheduled were rounded to 100%. Refill adherence was calculated based on the cumulative sum of days that a patient was late for ART pick-up appointments, divided by the total number of days over all such periods in the study. This calculated the percentage of time the patient was without medication over the whole study period. Children were categorized as either good adherence (≥95%) or poor adherence (<95%) [26,27,28].

### 2.2. Laboratory Testing

Children over 18 months old were tested by serology only, which classified children as infected or uninfected. Children under 18 months old were also initially tested serologically. After this, positive and negative samples from HIV-exposed children were tested for DNA by polymerase chain reaction (PCR) on dried blood spots. Those with viral DNA detected by PCR were classified as infected, while those with negative PCR results were classified as uninfected.

The COBAS^®^ AmpliPrep/COBAS^®^ TaqMan^®^ HIV-1 test (Roche Molecular Systems, Branchburg, NJ, USA) is a quantitative nucleic acid amplification test for the detection of HIV-1 RNA and TriTEST reagent (CD3/CD4/CD45) from Becton Dickinson BioSciences (San Jose, CA, USA) was used for the detection of CD4 count by flow cytometry in the BIDI.

In Thailand, viral load monitoring is performed annually, and CD4 level is performed every 6 months.

### 2.3. Statistical Analysis

All continuous data were compared with Student’s *t*-test and the Mann–Whitney *U* test as appropriate. Categorical data were compared with the Chi-square test. A *p*-value of <0.05 was considered to be statistically significant. The risk factors considered were sex, age, BMI, previous TB disease, parental TB history, adherence, last WHO stage, HAART, nadir CD4 cell counts, nadir hematocrit, peak cholesterol, peak triglyceride, hospitalization, pneumonia, ILI, OIs, study status, distance of residence from the clinic, caregiver and having a father as the main caregiver. Therefore, the sample size was calculated by a proportional formula, which assumed that the prevalence of non-suppressed virologic load was 7% among HIV-infected children [29]. Thus, the calculated minimum sample size for the dVL children was 25.

## 3. Results

There were 202 HIV-infected children in the BIDI. A total of 75 children in the prospective phase and 127 children in the retrospective phase were included. A total of 95 cases of the retrospective cases were excluded. In 77 cases, there was follow-up of less than three times a year (35 transferred to adult care, 13 transferred to other hospitals and 29 were loss to follow-up) There were also 15 cases of receiving ART for less than two years and three cases of no ARV.

**Figure 1:**
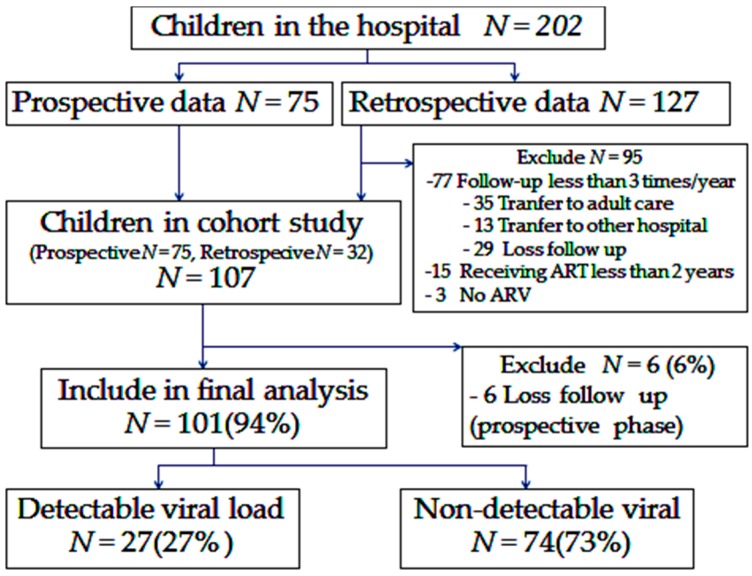
Schematic of patient flow of human immunodeficiency virus (HIV)-infected children in the study. ART—antiretroviral therapy; ARV—antiretroviral agent.

A total of 107 HIV-infected children were enrolled. Six children were excluded from the final analysis due to loss to follow-up. Thus, of the 107 children enrolled, 101 were included in the final analysis. Of these 101 children, 27 (27%) were classified as dVL, while 74 (73%) were non-dVL children (Figure 1).

Table 1 compares the demographics, and characteristics of dVL and non-dVL children who received ARV and close follow-up. The mean ages of dVL and non-dVL children were 12.0 and 12.3 years, respectively. Age, sex, BMI for age *z*-scores, previous TB disease history and parental TB history of both groups were not significantly different (*p* > 0.05).

The characteristics as well as clinical and laboratory factors of the study children during the study are shown in Table 2. The prevalence of adherence ≥95%, ILI and OIs were higher in dVL children compared with non-dVL children *(p* < 0.05). The mean lower nadir CD4 cell count during the study was higher in dVL than non-dVL children (646 compared to 867, respectively; *p* < 0.05). No significant differences between both groups were found in the last WHO stage, the prevalence of PI, peak cholesterol, peak triglyceride, hospitalization, pneumonia, study status, distance of residence from the clinic, caregiver and the prevalence of the father as the main caregiver (all *p* > 0.05). 

The associations between dVL children and factors, along with multivariable logistic regressions analysis, are shown in Table 3. In multivariable analysis, dVL was significantly associated with ILI (OR = 9.6, 95% CI = 1.34–69.36), the proportion of adherence (OR = 0.195, 95% CI = 0.047–0.811) and nadir CD4 level during the study (OR = 1.102, 95% CI = 1.100–1.305).

## 4. Discussion

The best prevention for ILI or influenza infection is the annual influenza vaccination [9]. This study demonstrated that dVL in HIV-infected children was significantly associated with ILI because high viral load may decrease the seroprotective effects of influenza vaccine. A previous study showed that poor seroprotection of the influenza vaccine was associated with high dVL (RNA > 40 copies/mL) in HIV-infected children [30]. In addition, Leahy et al. showed that a high proportion of RNA ≤ 40 copies/mL was associated with a low incidence of ILI, because children on HAART with full virologic suppression were more likely to sustain a robust serological response to the vaccine [31]. These findings suggest that influenza vaccination given during a virologic suppression increases the immunogenicity of the vaccine. However, the effectiveness of annual influenza vaccines for ILI varies from season to season and the data of seroprotection of trivalent influenza vaccine show that it often only spans an estimated six months [9,32].

Adherence to ART is an important determinant of treatment outcome. It could be argued that effective ART has made HIV a chronic disease and therefore issues affecting children living with HIV are similar to those affecting children with chronic diseases in general [33]. HIV-infected patients also require almost perfect levels of adherence to achieve long-lasting non-dVL with suboptimal adherence to ART being the most common cause of virologic failure [34]. In the present study, dVL was significantly associated with the poor adherence. This finding is similar to a previous study, which demonstrated that poor adherence increased the odds of virologic non-suppression (adjusted odds ratio = 3.4, 95% CI = 2.9–3.9) [35]. In addition, a previous study demonstrated that perfect adherence was associated with a higher probability of undetectable viral load at the 12-month visit (OR = 4.1, 95% CI= 1.8–9.1; *p* < 0.001) [36].

The present study showed that dVL in children was significantly associated with lower nadir CD4 level during the study. Similarly, a previous study demonstrated that measurements of viral load were strong predictors of CD4 cell decline over time among untreated HIV-infected individuals [37]. In practice, virologic failure occurs earliest, followed by immunological failure and clinical failure [38]. A lack of viral load monitoring can lead to delayed and unnecessary switches to new ART, promoting the development of resistance and limiting future treatment options [39,40].

Our study had several limitations. Firstly, viral load monitoring was performed annually and could not be re-evaluated during the study. Thus, some dVLs may have been temporary events. Secondly, there were missing data in the self-reporting of illness by parents. Another limitation was the duration of study because there was low incidence of epidemic influenza infection in Thailand during the study, which may have decreased the incidence of ILI cases. Thirdly, some diseases were diagnosed by clinical manifestations and were not confirmed diagnoses. Finally, the sample size was small.

In conclusion, the prevalence of dVL was 27%. In multivariable analysis, the prevalence of adherence <95% (poor adherence) and ILI were significantly higher in dVL than non-dVL children. The mean nadir CD4 cell count during the study was significantly lower in dVL than non-dVL children. Furthermore, dVL among HIV-infected children was associated with ILI, poor adherence and lower nadir CD4 during the study.

## Figures and Tables

**Table 1:** Demographics and characteristics of children at first visit.

Factor	Group A Detectable Viral Load (*N* = 27)	Group B Non-Detectable Viral Load (*N* = 74)	*p*-Value
Age (years)			0.695
Mean ± SD	12.0 ± 4.3	12.3 ± 4.3	
Sex, *n*			1.00
Male	14	37	
Female	13	37	
BMI for age *z*-scores (standard deviation scores)			0.100
Obesity (>+2 SD)	2	5	
Overweight (>+1 SD)	0	6	
Recommended (−2 SD to +1 SD)	20	46	
Thinness (<−2 SD)	4	15	
Severe thinness (<−3 SD)	1	2	
Previous TB disease history, *n* (%)	5 (18.5)	8 (10.8)	0.325
Parent TB history, *n* (%)	23 (85.2)	59 (79.7)	0.584

SD—standard deviation; BMI—body mass index; TB—tuberculosis.

**Table 2:** Clinical and laboratory factors as well as characteristics of study children.

Factor	Group A Detectable Viral Load (*N* = 27)	Group B Non-Detectable Viral Load (*N* = 74)	*p*-Value
Adherence ≥ 95%, *n* (%)	17 (63.0)	65 (87.8)	**0.008**
**Last WHO stage, *n* (%)**			
1, 2	20 (74.1)	47 (63.5)	0.353
3, 4	7 (25.9)	27 (36.5)	
**HAART, *n* (%)**			
PI	15 (55.6)	37 (50.0)	0.659
Non-PI	12 (44.4)	37 (50.0)	
Nadir CD4 cell count during the study, mean ± SD, cell/µL	646.0 ± 326.2	867.9 ± 341.1	**0.025**
Nadir hematocrit, mean ± SD, g/dL	37.1 ± 4.3	38.3 ± 3.1	0.222
Peak cholesterol, mean ± SD, mg/dL	186.0 ± 35.0	193.4 ± 37.6	0.478
Peak triglyceride, mean ± SD, mg/dl	135.8 ± 75.1	126.8 ± 59.0	0.608
Hospitalization, *n* (%)	7 (25.9)	15 (20.3)	0.590
Pneumonia, *n* (%)	5 (18.5)	18 (24.3)	0.604
ILI, *n* (%)	11 (40.7)	13 (17.6)	**0.020**
OIs, *n* (%)	12 (44.4)	16 (21.6)	**0.028**
**Study status, *n* (%)**			
Yes	22 (81.5)	63 (85.1)	0.759
No	5 (18.5)	11 (14.9)	
**Distance of residence, *n* (%)**			
Short	25 (92.6)	64 (86.5)	0.501
Long	2 (7.4)	10 (13.5)	
**Caregiver, *n* (%)**			
Parent	14 (51.9)	36 (48.6)	0.712
Relative	11 (40.7)	30 (40.5)	
Care Center	2 (7.4)	8 (10.8)	
Father as main caregiver, *n* (%)	3 (11.1)	10 (13.5)	1.00

Bold data: *p*-values < 0.05. WHO: The World Health Organization; HAART: highly active antiretroviral therapy; ILI—influenza-like illness; OIs—opportunistic infections.

**Table 3:** Factors associated with detectable viral load and multivariable logistic regressions analysis.

Significant Factors	Odds Ratio	*p*-Value	95% CI
ILI	9.629	0.025	1.337–69.359
Adherence	0.195	0.025	0.047–0.811
Lower nadir CD4 during the study	1.102	0.038	1.100–1.305
OIs	0.773	0.795	0.111–5.364

ILI—influenza-like illness; OIs—opportunistic infections; CI—Confidence Interval.
